# Sexual behaviour and risk factors for the acquisition of human papillomavirus infections in young people in Italy: suggestions for future vaccination policies

**DOI:** 10.1186/1471-2458-12-623

**Published:** 2012-08-07

**Authors:** Donatella Panatto, Daniela Amicizia, Cecilia Trucchi, Francesca Casabona, Piero Luigi Lai, Paolo Bonanni, Sara Boccalini, Angela Bechini, Emilia Tiscione, Carla Maria Zotti, Rosa Cristina Coppola, Giuseppina Masia, Angelo Meloni, Paolo Castiglia, Andrea Piana, Roberto Gasparini

**Affiliations:** 1 Department of Health Sciences, University of Genoa, Via Pastore, 1 16132, Genoa, Italy; 2 Department of Public Health, University of Florence, Florence, Italy; 3 Department of Public Health and Microbiology, University of Turin, Turin, Italy; 4 Department of Public Health, University of Cagliari, Cagliari, Italy; 5 Department of Biomedical Sciences, University of Sassari, Sassari, Italy

**Keywords:** Sexual behaviour, Human papillomavirus, Adolescents, Young people, HPV vaccination, Sexually transmitted diseases (STDs)

## Abstract

**Background:**

Human Papillomavirus (HPV) is the most common sexually transmitted infection. The main risk factors correlated with HPV infection are: early sexual debut, the number of partners, frequency and type of sexual contact and partner’s sexual histories.

We surveyed sexual habits among young people in order to provide information that might orient decision-makers in adopting HPV multi-cohort vaccination policies.

**Methods:**

We administered a questionnaire to students (14–24 years old) in five Italian cities.

**Results:**

7298 questionnaires were analyzed (4962 females and 2336 males); 55.3% of females (95% CI 53.9–56.7) and 52.5% of males (95% CI 50.5–54.5) reported regular sexual activity. The mean age at sexual debut was 15.7 ± 1.6 and 15.6 ± 1.6 for females and males, respectively, and the median age was 16 for both sexes.

With regard to contraceptive use during the last year, 63.6% of males and 62.8% of females responded affirmatively; 42.6% of males and 42.8% of females used condoms.

**Conclusion:**

The results reveal precocious sexual activity among respondents, with the mean age at first intercourse declining as age decreases. Condom use proved to be scant. Considering lifestyle-related risk factors, males appear to have a higher probability of acquiring HPV infection than females.

These data support the importance of promoting multi-cohort HPV vaccination strategies for females up to 25 years of age. It is essential to improve vaccination coverage through different broad-spectrum strategies, including campaigns to increase awareness of sexually transmitted diseases and their prevention.

## Background

Human Papillomavirus (HPV) is one of the most common sexually transmitted infections in sexually active adolescents and young people worldwide. HPV is generally spread through sexual contact [1] and direct skin-to-skin contact, the most common route of transmission being through penetrative sex [2].

All sexually active men and women are susceptible to acquiring genital HPV infection. The life-time risk of HPV infection is up to 80%. Most of these infections (up to 90%) are cleared within 2 years (12–24 months) and only a few become persistent [3]. The first HPV infection often occurs soon after the first sexual intercourse (FSI), the cumulative rate of genital HPV infection being approximately 50% over 3 years after the first intercourse [4,5]. The prevalence of HPV infection is highest among young women (18–24 years) soon after the onset of sexual activity, and falls gradually with age until the 4th-5th decade, when a second peak occurs for hormonal changes during perimenopause, followed by another decline [6]. Young subjects are exposed to HPV infection more often because of their sexual behaviour, and young women are more vulnerable than older women because the transformation zone is located on the ectocervix (cervical ectopy) [7].

The likelihood of developing precancerous lesions increases with persistent infections [8]. Persistent infection with an oncogenic type of HPV is a prerequisite to developing cervical cancer [9] and can determine cancer in other anatomical sites, such as the anus, vagina, vulva, oral cavity and oropharynx [10,11].

The most frequent risk factors correlated with the infection and persistence of HPV in the population are the following: initiation of sexual activity at a young age, the number of life-time sexual partners, frequency of sex or other intimate skin-to-skin contact, the sexual histories and behaviours of sexual partners, cigarette smoking, parity, the use of some types of oral contraceptives and alcohol consumption [2].

It is known that, among 150 different HPV types, only about 60 are able to infect the ano-genital area and that vaccination is a useful tool for preventing HPV infection caused by types more frequently involved [12-17]. For some years, two HPV vaccines have been on the market in Europe (EU): a quadrivalent vaccine (Gardasil®), licensed in September 2006, and a bivalent vaccine (Cervarix®), licensed in September 2007. Both vaccines have a prophylactic indication and are able to prevent pre-cancerous lesions (CIN II +) and cancers due to persistent infection with HPV-16 or HPV-18. Furthermore, both vaccines have been shown to elicit cross-protection against other high-risk HPV types [13-17]. Gardasil® also offers protection against genital warts caused by HPV-6 and HPV-11.

As HPV infection is exclusively contracted through sexual activity, it was recommended that adolescent females should be vaccinated against HPV before their sexual debut, as the main target population. Therefore, in March 2008, the Italian Ministry of Health recommended that regional authorities should start a campaign of free HPV vaccination for 12-year-old girls [18]. Since the introduction of HPV vaccination, many studies have been conducted with the aim of helping decision-makers choose the best vaccination policy [19-24].

Another important issue concerns the coverage rate, since only by achieving high coverage can circulation of the virus be rapidly reduced, thereby exerting a greater impact on diseases related to HPV genotypes. Indeed, in its HPV immunization plan, the Italian Ministry of Health has set the goal of achieving 95% coverage within 5 years of initiating the vaccination program in 12-year-old girls [18].

As many studies [2,25,26] of HPV prevalence and incidence indicate that the most consistent predictor of infection is sexual activity, particularly age on first intercourse and the number of sexual partners, we carried out a study of sexual habits among young people in Italy in order to provide information that might orient decision-makers in the choice of a multi-cohort vacci-nation policy; indeed, knowing the age of onset of sexual activity is essential to guiding Regional Health Authority recommendations regarding the optimal age for prophylactic HPV vaccination. The study did not envisage collecting data on vaccination acceptance.

## Methods

The study was approved by Ethic Committee of the Genoa Local Healthcare Unit, Italy (Azienda Sanitaria Locale 3 Genovese).

The study was carried out from May 2008 to May 2011.

### Questionnaire

We administered an *ad hoc* written questionnaire in order to survey the sexual behaviour and risk factors for HPV infections among young students (14–24 years old) in five Italian cities: Genoa, Florence, Turin, Cagliari and Sassari. The questionnaire comprised 20 questions co-vering the following specific items: demographics, level of education, sexual activity (penetrative genital-genital sex), age at first sexual intercourse, and sexual behaviour in terms of the number of sexual partners and contraceptive use (Additional file 1). The definition of regular sexual activity was to have sexual intercourse two or more times a month [27].

It took about 20 minutes to answer the self-administered questionnaire. In order to encourage openness and honesty in the answers, confidentiality and secrecy were assured by avoiding any questions regarding the identity of the respondents. During completion of the questionnaires, communication among the respondents was practically impossible, as the students were seated at a considerable distance from one another in spacious rooms. These precautions were taken in order to minimise the known limitations, and thus potential information bias, of self-reporting methods. Trained physicians were on hand to explain the questions, if necessary.

Before initiation of the study, the questionnaire was pilot tested on 50 Ligurian students, in order to evaluate the comprehension and relevance of our terms. As no problems were identified, the questionnaire was used in the study.

All questionnaires were checked on the basis of quality control (e.g. for internal coherence of the questionnaire).

### Study population

A representative sample ranging between 15% to 20% of secondary schools (high-schools, technical schools and vocational schools) located in the five above-mentioned cities participated in the study; these were randomly selected. A stratified sampling technique was used and strata were detected by age group (14–16 years, 17–19 and 20–24 years) and gender. From each school, about 10% of the students were interviewed. Furthermore, a random sample of university students (20-24-year age-group) stratified by gender was chosen from a list of those enrolled in the Universities of Genoa, Florence, Turin, Cagliari and Sassari.

As the primary target for vaccination against HPV is the female population, we recruited a higher number of females than males (2:1) in the study.

### Risk-groups

The subjects enrolled, independently of their age at the moment of filling in the questionnaire, were classified by risk-group in terms of their probability of acquiring HPV infection. The risk-groups were drawn up on the basis of literature data [28]. However, as no precise system of classifying subjects into risk groups is available in the literature, we distributed subjects on the basis of the following risk factors: age at first intercourse and lifetime sexual partners.

The low-risk group included subjects who had had only one partner in their life, regardless of their age at sexual debut, and those who had had 2 partners but had first experienced intercourse at > 15 years of age.

The medium-risk group comprised subjects who had had 2 partners and had begun sexual activity before the age of 15 years. This group also included subjects who had had 3 partners but whose sexual activity had begun at the age of ≥ 15 years.

Subjects reporting 3 partners and a sexual debut before the age of 14 years, and all subjects reporting ≥ 4 partners, regardless of age at sexual debut, were assigned to the high-risk group.

### Statistical analysis

Statistical analysis was performed by means of Statpages (Technical University of Denmark) [29], Openepi version 2.3.1 (Open Source Epidemiologic Statistics for Public Health) [30], Excel version 2011 14.1.3 (Microsoft Corporation – Redmond, Washington) and R (Development Core Team 2011) [31] software.

Differences between proportions were compared by means of z-test. Categorical variables were compared by Chi-square test and continuous variables by ANOVA. Variation across levels of single binomial proportions was tested by means of Chi-square test for trend. A p-value of ≤0.05 was considered statistically significant.

## Results

A total of 7500 students were invited to participate in the study; 51 declined. As few students refused to participate in the study, the reasons for refusal were not investigated.

The study involved 7449 volunteers aged 14–24 years (2386 males and 5063 females). We excluded 151 questionnaires from the analysis on the basis of quality control. Thus, 7298 questionnaires were analyzed (4962 females and 2336 males).

Out of 7298 subjects studied, 3334 (2222 females and 1112 males) were in the 14-16-year age-group, 2784 (1908 females and 876 males) were aged between 17 and 19 years, and 1180 (7832 females and 348 males) belonged to the 20–24 year age-group. Out of 7298 subjects studied, 6861 (4659 females and 2202 males) were Italians and 437 (303 females and 134 males) were foreigners.

Since the answers of the foreign students did not differ from those of the Italian students, a combined statistical analysis was performed. Likewise, as no statistically significant differences emerged among the answers given by the students from the five Italian cities, this statistical analysis was also combined.

A total of 55.3% of females (95% CI 53.9 – 56.7) and 52.5% of males (95% CI 50.5 – 54.5) reported regular sexual activity. No statistically significant differences were found on considering gender.

The study considered only subjects who declared regular sexual activity after their sexual debut. However, 21 females and 15 males who declared having had a sexual debut reported irregular sexual activity. These volunteers have been included in the group of subjects “NO regular sexual activity” (Table 1).

**Table 1:** Regular sexual activity of enrolled subjects, age at sexual debut and subjects with regular sexual activity prior 15th birthday by gender and age-group

Regular sexual activity** of enrolled subjects						
**SEXUAL ACTIVITY**	**FEMALES**			**MALES**		
	**Age-group (years)**			**Age-group (years)**		
	**14-16**	**17-19**	**20-24**	**14-16**	**17-19**	**20-24**
YES, N°(%),	689 (31.0)	1316 (69.0)	741 (89.1)	337 (30.3)	594 (67.8)	296 (85.0)
95% CI	29.1- 32.9	66.8 - 70.9	86.9 - 91.2	27.6 - 33.0	64.6 - 70.8	81.2 -88.7
NO, N°(%)	1414 (63.6)	485 (25.4)	65 (7.8)	645 (58.0)	203 (23.2)	26 (7.5)
95% CI	61.6 - 65.6	23.4 - 27.3	5.9 - 9.6	55.1 - 60.9	20.4 - 25.9	4.7 – 10.3
NR** , N°(%)	119 (5.4)	107 (5.6)	26 (3.1)	130 (11.7)	79 (9.0)	26 (7.5)
**Age at sexual debut**						
Mean ± SD	15.7 ± 1.6			15.6 ± 1.6		
Median	16			16		
(25^th^ -75^th^ P)	(15–17)			(15–17)		
	**14-16**	**17-19**	**20-24**	**14-16**	**17-19**	**20-24**
N°	688	1313	738	337	593	291
	NR*(1)	NR*(3)	NR*(3)	NR*(0)	NR*(1)	NR*(5)
Mean	14.5	15.7	16.9	14.3	15.8	16.7
SD	±1.0	±1.3	±1.8	±0.9	±1.3	±1.7
Median	15	16	17	14	16	17
25^th^ -75^th^ P	14-15	15-17	16-18	14-15	15-17	16-18
Subjects with regular sexual activity** prior to 15th birthday						
	**14-16**	**17-19**	**20-24**	**14-16**	**17-19**	**20-24**
Sexual debut < 15 year	327	257	60	203	108	26
	(47.5%)	(19.6%)	(8.1%)	(60.2%)	(18.2%)	(8.9%)
(95% CI)	(43.8-51.2)	(17.4-21.7)	(6.1-10.1)	(54.9-65.4)	(15.1-21.3)	(5.6-12.2)
Sexual debut ≥ 15 year	361	1056	678	134	485	265
	(52.5%)	(80.4%)	(91.9%)	(39.8%)	(81.8%)	(91.1%)
(95% CI)	(48.8-56.2)	(78.2-82.5)	(89.9-93.9)	(34.6-45.0)	(78.7-84.9)	(87.8-94.4)

* NR = non-responders.

** Definition of regular sexual activity: having sexual intercourse two or more times a month.

Table 1 shows regular sexual activity and age at sexual debut by age-group and gender. It also reports the sexually active subjects who stated regular sexual activity prior to their 15^th^ birthday. The percentage of subjects in each age-group who were sexually active before their 15^th^ birthday was calculated in order to better assess the changes in sexual habits among the young.

With regard to regular sexual activity, a significant (p < 0.001) increasing trend in proportions by age was observed in both sexes.

Among females, the mean age at sexual debut was 15.7 ± 1.6 and the median age was 16 (25^th^ and 75^th^ percentiles = 15-17). Among males, the mean age at sexual debut was 15.6 ± 1.6 and the median age was 16 (25^th^ and 75^th^ percentiles = 15-17).

Comparison of the mean ages at sexual debut among the age-groups of respondents of the same sex was carried out by means of analysis of variance (ANOVA). This revealed a highly significant difference (p < 0.001), indicating that, among the subjects who declared sexual activity, younger subjects debuted earlier than their older counterparts. Most females had their first sexual intercourse with a partner 2.4 years older, while males first had intercourse with a partner 0.5 years older (data not shown).

Table 2 shows the characteristics of sexual behaviour by gender and age-group among sexually active subjects. The number of lifetime sexual partners increases with age in both sexes, and males report more multiple partnerships than females. No difference was found in the number of partners prior to the respondent’s 15^th^ birthday, nor in the number of sexual partners in the last year among the different age-groups.

**Table 2:** Characteristics of sexual behaviour by gender and age-group among respondents who declared sexual activity

	**FEMALES**			**MALES**		
**Age-group (years)**	**14-16**	**17-19**	**20-24**	**14-16**	**17-19**	**20-24**
**Age of first sexual partner (years)**						
**N°**	668	1293	732	330	575	286
	(NR* = 21)	(NR* = 23)	(NR* = 9)	(NR* = 7)	(NR* = 19)	(NR* = 10)
**Mean age**	17.1	18.3	19.5	15.0	16.1	17.2
**SD**	2.2	2.73	3.43	1.67	2.39	3.07
**Range**	12-36	12-38	13-39	11-22	11-38	11-37
**Lifetime sexual partners (number)**						
**N°**	668 (NR* = 21)	1288 (NR* = 28)	733 (NR* = 8)	312 (NR* = 25)	548 (NR* = 46)	277 (NR* = 19)
**Median**	1	2	2	2	3	3
**25**^**th**^**-75**^**th**^**P**	1-2	1-3	1-4	1-3.75	1-5	2-7
**Range**	1-30	1-32	1-50	1-27	1-27	1-32
**Sexual partners prior to 15**^**th**^**birthday (number)**						
**N°**	319 (NR* = 8)	255 (NR* = 2)	60 (NR* = 0)	192 (NR* = 11)	105 (NR* = 3)	25 (NR* = 1)
**Median**	1	1	1	1	2	1
**25th -75th P**	1-2	1-2	1-2	1-3	1-3	1-3
**Range**	1-20	1-15	1-15	1-16	1-10	1-5
**Sexual partners in the last year (number)**						
**N°**	666 (NR* = 23)	1286 (NR* = 30)	736 (NR* = 5)	324 (NR* = 13)	571 (NR* = 23)	282 (NR* = 14)
**Median**	1	1	1	1	1	1
**25th-75th P**	1-1	1-1	1-1	1-2	1-2	1-2
**Range**	1-10	1-19	1-13	1-21	1-15	1-15

* NR = non-responders.

**Figure 1:**
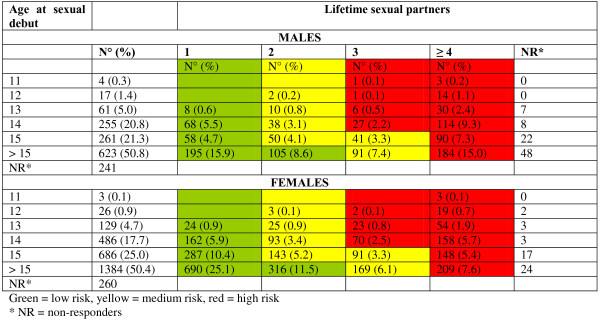
Number of males and females in the various HPV risk-groups.

Figure 1 reports the numbers and percentages of males and females, broken down according to the risk of acquiring HPV infection. Among males, 434 (38.2% 95%CI 35.8-40.5), 232 (20.4% 95%CI 18.4-22.3) and 470 (41.4% 95%CI 39.9-43.8) subjects fell into the low-risk, medium-risk and high-risk groups, respectively. Among females, 1479 (55.0% 95%CI 53.1-56.9), 524 (19.5% 95%CI 18.0-20.9) and 686 (25.5% 95%CI 23.8-27.1) subjects fell into the low-risk, medium-risk and high-risk groups, respectively.

Table 3 shows the percentage of subjects who used a contraceptive method during their sexual debut by gender and age-group. Overall, the use of a contraceptive method at sexual debut was reported by 62.2% (95% CI 59.5-64.9) of males; 58.9% (95% CI 56.1-61.6) indicated using a condom. Among females, 70.3% (95% CI 68.6-72.0) reported using a contraceptive method at sexual debut; 67.2% (95% CI 65.9-69.3) specified using a condom. Moreover, a significant (females: p < 0.001; males: p < 0.05) increasing trend in proportions of the use of contraceptive methods by age was observed in both sexes.

**Table 3:** Use of a contraceptive method during first intercourse and during last year among respondents who declared sexual activity by gender and age-group

	**FEMALES**			**MALES**		
**Use of a contraceptive method at sexual debut**						
	**Age-group (years)**			**Age-group (years)**		
	**14-16**	**17-19**	**20-24**	**14-16**	**17-19**	**20-24**
YES* , N°(%)	447/689 (64.9)	914/1316 (69.4)	571/741 (77.0)	183/337 (54.3)	398/594 (67.0)	182/296 (61.4)
95% CI	61.3-68.7	66.9-71.9	74.0-80.0	49.0-59.6	63.7-70.3	55.9-66.9
YES CONDOM, N°(%)	435/689 (63.1)	870/1316 (66.1)	541/741 (73.0)	178/337 (52.8)	372/594 (62.6)	173/296 (58.4)
95% CI	59.5-66.7	63.5-68.7	69.8-76.2	47.5-58.1	58.7-66.5	52.8-64.0
NR**, N°(%)	68/689 (9.9)	112/1316 (8.5)	90/741 (12.1)	41/337 (12.2)	76/594 (12.8)	40/296 (13.5)
**Use of a contraceptive method during last year**						
	14-16	17-19	20-24	14-16	17-19	20-24
YES* + , N°(%)	324/543	850/1223	375/700	132/265	369/524	171/267
	(59.6)	(69.5)	(53.5)	(49.8)	(70.4)	(64.0)
95% CI	55.5-63.7	66.9-72.1	49.8-57.2	43.8-55.8	66.5-74.3	58.2-69.7
YES + CONDOM, N°(%)	244/543	554/1223	257/700	91/265	244/524	115/267
	(45.0)	(45.3)	(36.7)	(34.3)	(46.5)	(43.0)
95% CI	40.8-49.2	42.5-48.1	33.1-40.3	28.6-40.0	42.2-50.8	37.1-48.9
YES PILL, N°(%)	66/543	300/1223	157/700			
	(12.1)	(24.5)	(22.4)			
95% CI	9.3-14.8	22.1-26-9	19.3-25.5			
NR**, N°(%)	63/543 (11.6)	106/1223 (8.7)	126/700 (18.0)	64/265 (24.1)	68/524 (13.0)	39/267 (14.6)

* condom, pill, coitus interruptus.

** NR = non-responders.

+ every time, almost every time.

With regard to the use of contraceptive methods during the last year, 63.6% (95% CI 60.7-66.5) of males responded affirmatively, and 42.6% (95% CI 39.6-45.6) reported using a condom. Among females, 62.8% (95% CI 60.9-64.7) responded affirmatively: 42.8% (95% CI 40.8-44.7) reported using a condom and 21.2% (95% CI 19.6-22.8) oral contraception.

With regard to the use of a contraceptive method during the last year by gender and age-group, an increasing trend (p < 0.05) was observed by age for males (Table 3).

## Discussion

This paper reports the results of a study of self-reported sexual behaviour in adolescents and young people aged 14–24 years in Italy.

More than two thirds of the students involved in this study were females, who constitute the target population of HPV vaccination. The results yielded by this sample therefore enabled the suitability of the current vaccine-tion strategies in Italy to be evaluated indirectly, in that sexual activity is the most consistent predictor of the probability of acquiring HPV infection.

Exposure to HPV infection is determined by risks factors such as an early sexual debut, a high number of lifetime sexual partners, a high number of recent or current sexual partners and the sexual histories and behaviours of sexual partners [2,25]. Condom use has a protective effect, although its effectiveness in reducing HPV acquisition has not been completely clarified [32,33].

The results of our study highlight the early start of sexual activity among young students in Italy, with a mean age at sexual debut of 15.7 ± 1.6 for females and 15.6 ± 1.6 for males. No statistically significant differences were found on considering gender and the city of residence. In recent years, lifestyle changes, especially in sexual behaviour, have been registered among adolescents in Europe [25,34]. Young people travel a lot and have more open and changeable interpersonal relationships. We noted a decrease in the mean age at first intercourse on comparing the various age-groups of the subjects (Table 2); indeed, in the 14–16 age-group, 47.5% of the sexually active females and 60.2% of the sexually active males reported having their first sexual intercourse before their 15th birthday, and a third of the interviewees aged 14–16 years old declared that they already indulged in regular sexual activity.

Most of the females stated that their sexual debut was with a partner 2.4 years older, while the males reported their first intercourse with a partner of the same age. As the sexual behaviour of the partner, particularly the number of previous sexual partners, is a risk factor for the transmission of mucosal HPV infection, having an older partner increases the probability of acquiring the virus. Consequently, females could have a higher risk than males at sexual debut.

The number lifetime sexual partner increases with age, and males report more multiple partnerships than females.

On the basis of age at sexual debut and the number of lifetime sexual partners, the respondents were grouped into different risk-group. Considering lifestyle-related risk factors, males appear to have a higher probability of acquiring HPV infection than females. Indeed, more males fell into the high-risk group (41.4%) than the low- (38.2%) or medium-risk groups (20.4%), while most females were in the low-risk group (55.0%). The exact probability of transmission through sexual contact or partnership is not precisely known. Barnabas et al. estimated a value of transmission probability using a mathematical modelling analysis. They considered that the transmission probability could varied between 0 to 1, and they found that the probability of transmission was approximately 0.4 for annual partner change rate [28]. Burchell et al. report a partnership transmission probability of 0.20; they observe little difference in the rate of male-to-female (3.5 transmissions per 100 person-months) versus female-to-male transmission (4.0 transmissions per 100 person-months) [35]. Other studies have found higher rates of female-to-male than male-to-female transmission [36-38].

Our data indicate a low use of condoms. With regard to regular condom use during the last year, no difference between the genders emerged. In the female group, however, we noted statistically significant differences (p < 0.05) between age-groups. The percentage of females who regularly use condoms was seen to decline as age increased, in particular in the 20–24 age-group. This could be explained by the fact that older females reported more stable and durable relationships and a greater use of the pill. We surmise, also on the basis of literature data [39,40], that many young people use condoms to prevent unwanted pregnancies, but that they have scant knowledge or perception of the risk of sexually transmitted infections. In the male group, the percentage of students who regularly use condoms increased with increasing age.

Direct comparison of our results with data from other studies conducted in Italy and other European countries is complicated by the different methodologies used and the different populations monitored. A study published in 2006 reported data on sexual behaviour in 59 countries worldwide during the period 1996–2006. The authors found median ages at sexual debut of 17.5 and 18.5 years among young men and women, respectively, in Italy. The difference between these findings and those of our study can be attributed to the cohorts of the subjects interviewed; our students were born between 1984 and 1996, while those of Wellings’ study were born between 1965 and 1969 [41]. This confirms a lifestyle change in recent years. In a survey conducted in 2006–2007 in 7 European countries, Crochard et al. reported a median age at sexual debut of 16 and 17 for males and females, respectively, in Italy [25]. Our research group carried out an investigational study on sexual behaviours among adolescents in a Region of Northern Italy from 2006 to 2007, and found similar results [42].

## Conclusions

These data confirm the importance of promoting multi-cohort HPV vaccination strategies for young females. The strategy of free vaccination for females up to 25 years of age in Italy appears advantageous. This approach would rapidly reduce circulation of the virus and, consequently, have a greater impact on diseases related to HPV genotypes at high oncogenic risk.

Furthermore, it is essential to make every effort to improve the coverage of HPV vaccination. Indeed, data on coverage in Italy (updated as of 30^th^ June 2011) revealed a coverage rate of 65% for the primary target of vaccination (12-year-old girls); since the beginning of the immunization program, no improvement has yet been observed [43]. To this end, it is necessary to implement a range of broad-spectrum strategies, including communication campaigns to increase awareness of sexually transmitted infections and their prevention. Teenagers’ sexual behaviours have both short-term and long-term consequences, and interventions that focus on this target population may be the most effect-tive in helping to promote overall health among young adults.

Information campaigns can only be successful if they reach their target populations. In the present case, as our target population is that of young people, we should exploit the means of communication which they themselves use, such as social networks (facebook, twitter) and websites dedicated to the young (information online). To achieve high coverage rates of anti-HPV vaccination, these channels of communication have been utilised in the United Kingdom, where very good results have been obtained within a short time [44].

Finally, it is very important that everybody understand that safe and efficacious vaccines for the prevention of cervical cancer are available and provide important individual and public benefits.

## Abbreviations

HPV: Human papillomavirus; FSI: First sexual intercourse; NR: Non-responders; CI: Confidence interval; STDs: Sexually Transmitted Diseases.

## Competing interest

The authors declare that they have no competing interests.

## Authors’ contributions

RG supervised the research; RG and DP designed the study and coordinated the Genoa unit research; PLL optimized the informatics database; RG, DP, PLL, PC, AP performed the statistical analyses and evaluated the results; DP and DA wrote the manuscript; PB, RCC, PC, CMZ coordinated the data collection in Florence, Cagliari, Sassari and Turin and performed the final local quality control; CT, FC, SB, AB, ET, GM, AM, AP collected data and made the first local quality control; All Authors revised the manuscript and gave their contribution to improve the paper; All authors read and approved the final manuscript.

## Pre-publication history

The pre-publication history for this paper can be accessed here:

http://www.biomedcentral.com/1471-2458/12/623/prepub

## Supplementary Material

Additional file 1 Questionnaire. Click here for file
