# Supplementary material for: Sexual behaviour and risk factors for the acquisition of human papillomavirus infections in young people in Italy: suggestions for future vaccination policies
Source: BMC Public Health. 2012 Aug 7;12:623. doi: 10.1186/1471-2458-12-623 (PMC3490840; doi:10.1186/1471-2458-12-623)
Supplement: Additional file 1 — Questionnaire. [file 1471-2458-12-623-S1.doc]

**QUESTIONNAIRE**

Code ___________

**SOCIO-DEMOGRAPHIC CHARACTERISTICS**

Sex ☐M

☐F

Nationality __________________

Age _______

Residence city/town ____________

Type of family: ☐ Two biological parents

☐ Two biological parents and brothers/sisters

☐ Stepfamily

☐ Single parent

☐ Other

☐Non-responder

Type of school you are attending: ☐University

☐High-school

☐Technical school

☐Vocational school

☐Non-responder

**SEXUAL BEHAVIOUR**

Have you ever had penetrative genital-genital sex?

☐Yes

☐No

☐Non-responder

Age when you had your first sexual experience: ______________ ☐Non-responder

Age of your first sexual partner: _____________ ☐Non-responder

Do you have regular sexual activity?

☐Yes

☐No

☐Non-responder

Have you had regular sexual activity in the last year?

☐Yes

☐No

☐Non-responder

Did you start regular sexual activity before or after you were 15 years old?

☐Before

☐After

☐Non-responder

How many sexual partners have you had in your life? (indicate a number) _____________________☐Non-responder

How many sexual partners did you have prior to your 15^th^ birthday? (indicate a number) _________☐Non-responder

How many sexual partners have you had in the last year? (indicate a number) __________________☐Non-responder

Did you use any contraceptive method during your first sexual intercourse?

☐Yes

☐No

☐Non-responder

If yes, what contraceptive method did you use?

☐Condom

☐Pill

☐Coitus interruptus

☐Other

☐Non-responder

Have you used any contraceptive method in the last year?

☐Every time

☐Almost every time

☐Rarely

☐Never

☐Non-responder

If you did, what contraceptive method have you used?

☐Condom

☐Pill

☐Coitus interruptus

☐Other

☐Non-responder

How often have you used a condom in the last year?

☐Every time

☐Almost every time

☐Rarely

☐Never

☐Non-responder

How often have you used the pill in the last year?

☐Every time

☐Almost every time

☐Rarely

☐Never

☐Non-responder
